# Optimizing Alignment Parameters During Craniocervical Stabilization and Fusion: A Technical Note

**DOI:** 10.7759/cureus.7160

**Published:** 2020-03-02

**Authors:** Fraser Henderson, Robert Rosenbaum, Malini Narayanan, John Mackall, Myles Koby

**Affiliations:** 1 Neurological Surgery, University of Maryland Prince George's Hospital Center, Largo, USA; 2 Neurological Surgery, Doctors Community Hospital, Lanham, USA; 3 Neurological Surgery, The Metropolitan Neurosurgery Group, Silver Spring, USA; 4 Neurological Surgery, University of Maryland Prince George’s Hospital Center, Cheverley, USA; 5 Neurological Surgery, D&K Medical, LLC., Lanham, USA; 6 Radiology, Doctors Community Hospital, Lanham, USA

**Keywords:** clival-axial angle, craniocervical fusion, harris measurement, grabb-oakes measurement, dynamic imaging, craniocervical alignment, craniocervical reduction, gaze angle, mandible-axial angle, orbital-axial angle

## Abstract

Proper craniocervical alignment during craniocervical reduction, stabilization, and fusion optimizes cerebrospinal fluid (CSF) flow through the foramen magnum, establishes the appropriate “gaze angle”, avoids dysphagia and dyspnea, and, most importantly, normalizes the clival-axial angle (CXA) to reduce ventral brainstem compression. To illustrate the metrics of reduction that include CXA, posterior occipital cervical angle, orbital-axial or “gaze angle”, and mandible-axial angle, we present a video illustration of a patient presenting with signs and symptoms of the cervical medullary syndrome along with concordant radiographic findings of craniocervical instability as identified on dynamic imaging and through assessment of the CXA, Harris, and Grabb-Oakes measurements.

## Introduction

Craniocervical reduction, stabilization, and fusion are performed after suboccipital craniotomy for Chiari I malformation with basilar invagination, craniocervical instability due to trauma or neoplasm, and connective tissue disorders [1-3]. Correct craniocervical reduction/realignment is of paramount importance. Proper spinal alignment optimizes cerebrospinal fluid (CSF) flow through the foramen magnum, establishes the appropriate “gaze angle”, avoids dysphagia and dyspnea, and, most importantly, normalizes the clival-axial angle (CXA) to reduce ventral brainstem compression [2,4-7]. The authors introduce a direct measure of gaze angle: the orbital-axial angle (OAA) and a mandible-axial interval (MAI) metric of the pharyngeal space to avoid dyspnea and dysphagia.

To illustrate the metrics of reduction, we present a patient with signs and symptoms of the cervical medullary syndrome, which included severe head and neck pain, spasticity, weakness, sensory loss, and radiological indices of ventral brainstem compression, a kyphotic CXA, craniocervical instability, along with pathological translation between flexion and extension images [4,7-9]. A CT scan demonstrated rotary C1-C2 subluxation (Fielding Type 1) upon full neck rotation [10]. After a standard exposure, instrumentation was placed in the manner of Goel and Harms.

The reduction is an iterative process. Orthogonality is first established between the occiput, C1, and C2 to establish sagittal and coronal alignment. Traction with posterior translation and extension is then employed to achieve a CXA of >140°, reduce the medullary kink, and eliminate ventral brainstem compression. The reduction should accomplish OAA (“gaze angle”) of 95-105° to avoid either star gazing or downward gaze. The MAI is established at 10-20 mm, to avoid dysphagia and dyspnea. In the authors’ experience, attention to precise reduction improves the outcome [7,11,12].

## Technical report

The craniocervical reduction metrics and surgical techniques are discussed and demonstrated in the accompanying surgical video (Video 1). Of particular importance to craniocervical fusion and stabilization is the realignment of the cranium over C1 and C2. The goal is not only to normalize alignment but to reestablish a “gaze angle” appropriate to the individual and a jaw position that will not interfere with swallowing or breathing. To illustrate reduction, we present a chronically disabled patient with severe head and neck pain who presented with symptoms of cervical medullary syndrome, including hyperreflexia, weakness, sensory deficits, Romberg sign, and inability to perform tandem gate. Both CT scan and upright flexion/extension MRI demonstrated a kyphotic CXA of 120°, considered pathological since it was less than 135°, raising concern for abnormal stretching of the brainstem, out-of-plane loading, and pathological deformative stress. The Grabb-Mapstone- Oaks measurement, also known as the pB-C2 measurement, was 11 mm, exceeding the pathological threshold of 9 mm, and constituting ventral brainstem compression. The Grabb-Mapstone-Oaks measurement was taken from the dura to a line drawn from the basion to the posteroinferior C2 vertebrae. The Harris measurement was 12 mm, meeting the Harris criteria for instability. The Harris measurement is taken from the basion to the posterior axial line, and the vertical Harris measurement from the basion to the tip of the odontoid; a measurement of 12 mm or more constitutes instability. This patient presented with an interval of >12 mm, which reduced on extension to 7 mm, representing 5 mm of horizontal translation and exceeding the pathological threshold of translation (1-2 mm), further supporting the diagnosis of craniocervical instability. Rotational CT scan demonstrated 45° rotation of C1 upon C2 on full rotation to each side, constituting a diagnosis of atlantoaxial instability. The patient failed six months of conservative therapy and was considered a candidate for open reduction to restore normal craniocervical relationships followed by fusion-stabilization (Video 1).

**Video 1:** Technique of craniocervical reduction, stabilization, and fusion

The surgical procedure involves the placement of a Mayfield head clamp and a neck brace. The pins are kept free of the temporalis muscle to minimize postoperative pain. Care is taken to avoid any underlying hardware, arteries, or nerve. The patient is then turned and the neck brace is removed. Traction is applied and the Mayfield affixed. A fluoroscopic image is taken to assess orthogonality. Indices of orthogonality include an overlap of the occipital condyles and the mandibles, as well as the vertebral artery foraminae at C1.

During positioning, the patient is extended at the cervicothoracic junction and flexed slightly at the craniocervical junction to improve exposure. The patient is flexed slightly at the waist and knees to relax the spinal cord and the cauda equina. The patient is prepped and draped. The iliac crest is also prepped and draped to harvest bone marrow. The wound is injected generously with bupivacaine and epinephrine. Sensory evoked potentials and brainstem monitoring are performed.

Tricortical iliac crest allograft is soaked in saline for an hour before placement. The bone graft is a strip graft from the right iliac crest. The subocciput is exposed in the usual manner. Deep retractors are placed. The CT scan is then carefully evaluated and the lateral masses of C1 and pedicles of C2 are examined for the planning of the C1 and C2 screws. A small ruler is cut to the length, usually 23 mm, to measure from midline to the screw insertion site at the C1 lateral mass. After careful sub-periosteal dissection of the venous plexus away from the inferior edge of the C1 ring and lateral mass, a diamond burr is used to create a 2-mm pilot hole on the posterior surface of the C1 lateral mass. Then a hand or power drill is directed medially 8° and toward the anterior tubercle of C1 as seen on sagittal fluoroscopy, to a depth of 24 mm but not beyond the level of the posterior cortex of the anterior tubercle, as seen on the sagittal fluoroscopy. 

At the C2 level, holes are drilled angling 30° medially and superiorly: parallel to the pars interarticularis, and parallel to the C1 screws. This brings the screws supero-medially over the vertebral artery foraminae. A fluoro-CT is routinely performed to assess the appropriate positioning of the C1 and C2 screws. The odontoid should lie precisely centered between the lateral masses of C1.

**Figure 1:**
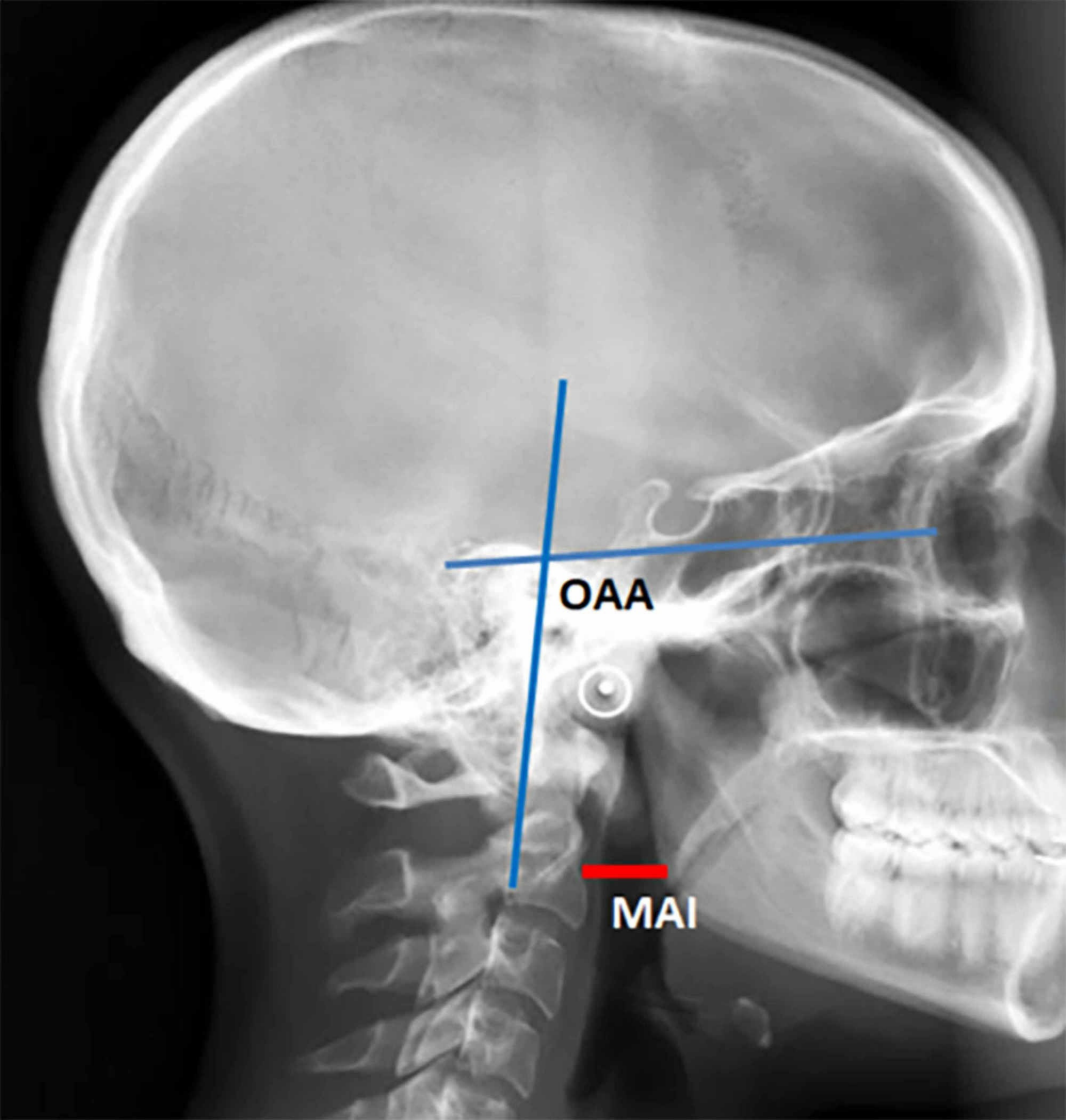
Orbital-axial angle and mandible-axial interval marked on a lateral skull X-ray The orbital-axial angle (OAA) is that angle subtended by a line from the mid-orbit tangential to the base of the pituitary fossa and the posterior axial line. The OAA should approximate 100° (95-105°). The mandible-axial interval (MAI) is a direct measure of the retropharyngeal space, optimally between 10 and 20 mm

The reduction portion of the surgery is very important. It involves traction, posterior translation, and extension. It is an iterative process. While the surgeon grasps the Mayfield, a colleague releases its clamp. Applying traction and reduction, we establish a CXA of >140°, an MAI of 10-20 mm, an OAA of 95-105°, and a posterior occipital cervical angle (POCA) of 95-105°. The OAA is subtended by a line from the mid-orbit tangent to the pituitary fossa and the posterior axial line. Ideally, this should equal the occipital-cervical angle. The MAI should be established at 10-20 mm to avoid dysphagia. The position of the mandible, and hence the MAI, is influenced by the degree of mouth opening; “optimal MAI” of 10-20 mm assumes a 15-mm bite block. A larger bite block will result in an unacceptable subluxation of the mandible, a more posterior position of the mandible, and thereby underestimation of the MAI (Figure 1).

A craniocervical stabilization device is selected and trialed over the subocciput (Cranial Fusion System; Life Spine, Huntley, IL). Screws are loosened for later insertion of the rods in the smooth and low-profile system preferred by our group. The subocciput is flattened with a Leksell and high-speed drill, and some decortication is performed over the subocciput. A line is drawn along midline so that the fixator is positioned precisely on the midline. The fixator is trialed and rods are then cut and bent to shape.

A bone graft, previously soaked in saline, is then drilled to engage the aperture of the craniocervical device superiorly and the spinous process and lamina of C2 and the ring of C1. The rods are then cut and bent to conform to the craniocervical angle. The rods are bent symmetrically. The rods are placed into the lateral cuniculi (cylindrical slots) of the craniocervical system and the system is trialed again. The bone marrow is injected into the tricortical iliac crest graft to improve fusion characteristics. The entire assembly is placed into the exposed craniocervical junction. 

The system is tapped down tightly to secure the allograft between the fixator and the C2 spinous process, and then the fixator is anchored to the subocciput with central 9-mm or 10-mm screws and two lateral 6-mm screws. Additional bone marrow and bone dust are packed around the edges to augment fusion. Here the graft can be seen tightly positioned between the aperture of the fixator and the C1 and C2 spinous process. A final fluoroscopic image is taken.

## Discussion

We report here in text and video the technical nuances necessary for the optimization of the craniocervical reduction portion of a stabilization and fusion surgery. Specifically, open reduction during craniocervical stabilization and fusion should include reduction of medullo-spinal deformity, normalization of “gaze angle”, and maintenance of appropriate pharyngeal space to optimize airway and swallowing.

Dynamic imaging

Dynamic imaging in flexion-extension is often necessary to demonstrate basilar invagination, craniocervical instability, and atlantoaxial instability [7,9,12]. Dynamic imaging demonstrated craniocervical instability in 9 of 29 patients with Down syndrome, and Menezes’ series of 100 children with Down syndrome demonstrated 24 subjects with craniocervical instability and 34 with C1-2 instability [13]. Odontoid instability and connective tissue disorders such as rheumatoid arthritis, Morquio, Marfan, and Ehlers-Danlos syndromes may be associated with pathological instability that is only revealed on flexion-extension imaging, especially in cases with a retroflexed odontoid.

The clival-axial angle

The CXA, the angle between the posterior axial line and the clival line, is a surrogate measure of anatomic brainstem deformity and the potential indicator for reduction/stabilization of the craniocervical junction [14]. Variously identified as the clivus-vertebral angle, the clivus-canal angle, and the clivus-cervical angle, the CXA is composed by the clivus line drawn along the lower third of the clivus to the top of the odontoid process and by the posterior axial line that is drawn along bone contour of the axis on CT (the “boney CXA”) or the ligamentous margin of the odontoid (the “soft tissue CXA”). 

The CXA in the neutral position is 158.2° ±9.8° in normal adults. The normal CXA in the neutral position may be based upon an X-ray or fluoroscopic image, an MRI ( the “soft tissue CXA”), or a CT scan (the “boney CXA”). These measurements will differ slightly by several degrees. Flexion of the neck decreases the CXA by 10°; extension increases the CXA by 10°. Botelho’s series of subjects with basilar invagination found a mean CXA of 120° (range 79-145°) [15]. A kyphotic CXA has been described as a “non-traditional basilar invagination” [5]. Neurological deficits were variably attributed to a CXA of less than 150° and 125° [3,14]. Kubota reported on a series of Chiari I malformation subjects, where the syringomyelia failed to resolve in those patients with a kyphotic CXA (<130°) [16].

A CXA of <135° is considered kyphotic; it results in medullary kinking and is inversely related to the increase in biomechanical stress of the brainstem or upper spinal cord [11]. A kyphotic CXA results in brainstem lengthening with resultant increased stress. Sawin and Menezes described the “fulcrum effect in basilar invagination, by which traction is applied to the caudal brainstem and rostral cervical spinal cord, producing prominent bulbar dysfunction and myelopathy” [17]. 

Correction of the CXA is associated with significant improvement of outcomes [1,11,12]. A kyphotic CXA is usually associated with platybasia. Platybasia is defined by the flattening of the basal angle, which is the angle between the anterior fossa and the line drawn along the posterior margin of the clivus; as the basal angle becomes more flattened and pathological, the corresponding CXA becomes more kyphotic [18]. Pang notes that platybasia must “necessarily narrow the clivus canal angle”, and that this “underlies most forms of basilar invagination, especially those with a retroflexed dens” [18]. Platybasia with encephalo-myelopathy from medullary kink is often found with degenerative conditions such as rheumatoid arthritis, inherited conditions such as Ehlers-Danlos syndrome, and other conditions including those in which bone weakening and subsequent platybasia and basilar invagination occur [12].

The Harris measurement

In addition to the kyphotic angulation of the brainstem, horizontal translation of the cranium between the occiput and atlas may occur. Cranial settling, which can occur with connective tissue disorders, may invite inordinate aggregate translation and subsequent basilar invagination with compression of the spinal cord or medulla.

The basion axis interval (BAI), or horizontal Harris measurement, is the distance from basion to the posterior axial line (PAL). The vertical Harris measurement is the interval from basion to the tip of the odontoid. Harris found that, in a large series of patients, a BAI of ≥12 mm or a vertical basion-to-odontoid measurement of ≥12 mm on standard X-ray represented instability [8]. Allowing a 20% magnification on the standard X-ray, this measurement would correspond to 10 mm on MRI or CT. However, a 12-mm rule has been adopted [9,12,19].

There should be no measurable translatory movement (sliding movement) between flexion and extension. A change in the horizontal Harris measurement of >1 mm, as measured in flexion and extension images, represents pathological translation between the basion and odontoid [9,11]. Therefore, flexion and extension measurements are useful to determine if there is pathological translation. Neurological improvement is associated with craniospinal alignment and the elimination of unstable translatory craniocervical movement [11,12]. 

The Grabb, Mapstone, and Oakes measurement

The Grabb-Oakes measurement, or pB-C2, is the interval from the dura perpendicular to the BC2 line (drawn from basion to posterior inferior C2 vertebra). A measurement of ≥9 mm suggested a high risk of ventral brainstem compression and was statistically correlated with clinical outcome. Oakes and colleagues showed that the pB-C2 measurement was statistically reproducible (p: <0.01), and that it was a “direct measurement of encroachment by the odontoid into the foramen magnum and spinal canal.” It was felt that this encroachment was more often the cause of brainstem compression in patients with Chiari malformation. Moreover, a pathological measurement (>9 mm) tends to be progressive [4]. 

Open reduction 

The goal is to establish the most comfortable and salutary position of the cranium with respect to the skull. This involves gentle traction, reduction, and, to the extent possible, posterior translation to bring the odontoid over the midpoint of the odontoid process. 

Extension of the craniocervical junction is employed to establish a CXA of >140°, which is associated with neurological improvement [3,5,7,11, 4,16]. However, the subject should not be excessively extended, as this may cause “star gazing”. In order to optimize the gaze position, the authors devised the gaze angle or orbito-axial angle (OAA). The OAA is that angle subtended by a line drawn from the mid orbit (approximately parallel to the hard palate) to the floor of the pituitary fossa and the posterior axial line. Generally, the OAA is optimal at 100° (Figure 1). It should be slightly more for a subject of shorter stature (who more often is looking upward), and less for a tall person (who is habitually looking downward). To reduce sub-axial stress, the OAA should approximate the POCA. The POCA is that angle subtended by the tangent to the flat posterior aspect of the occiput between the opisthion and occipital protuberance, and a line drawn along the posterior aspect of the third and fourth cervical facets. An increased POCA reflects a downward head tilt and is associated with increased adjacent segment degeneration due to increased biomechanical stresses [20].

In addition to the above measurements, the authors directly measured the distance from the mandible to the upper cervical spine, to ensure that the depth of the upper pharynx was optimal for breathing and swallowing. In the authors’ experience, a small distance from the angle of the mandible to the anterior axial line (<10 mm) or an excessive mandible-axial interval (>25 mm) results in dysphagia (Figure 1). Izeki et al. warn that a careless reduction may result in persistent dysphagia or life-threatening dyspnea [6].

A final consideration, in terms of alignment, is governed by a coronal view of the craniocervical spine or intraoperative CT, to assess the mid-position of the odontoid between the lateral masses of C1. In addition to the obvious biomechanical consequences of partial subluxation, translation to one side may result in obstruction of CSF flow or alteration of vertebral artery or peri-vertebral venous flow. 

The authors devoted a great deal of time with the alignment/reduction portion during craniocervical fusion in order to optimize the various measurements discussed above. The metrics must to some extent be individualized, reflecting both the stature and the anticipated type of activity of the patient. The thoughtful application of these concepts upon the authors’ cohort of subjects undergoing craniocervical stabilization was reflected in the lack of complaints regarding neck position postoperatively [12].

## Conclusions

Thoughtful reduction restores optimal craniocervical alignment and may eliminate basilar invagination and medullary kyphosis during craniocervical stabilization and fusion. Preoperative dynamic imaging demonstrates the potential and degree to which reduction may be accomplished intraoperatively. Normalizing the CXA appears to be of great importance. The OAA has been introduced as a direct measurement of the “gaze angle”, and the MAI is presented as a direct measurement of the pharyngeal space to optimize swallowing and breathing.
